# Adenomyoma/adenomyomatosis-associated mural intracholecystic neoplasms: analysis of clinico-pathologic, imaging, and molecular features of a consecutive case series

**DOI:** 10.1007/s00428-025-04077-7

**Published:** 2025-03-21

**Authors:** Alessandro Vanoli, Erica Travaglino, Marco Minetto, Anna Gallotti, Federica Grillo, Salvatore Corallo, Marcello Maestri, Andrea Peri, Paola Fugazzola, Francesca Antoci, Roberta Riboni, Antonio Di Sabatino, Luca Ansaloni, Andrea Pietrabissa, Gioacchino D’Ambrosio, Marco Paulli

**Affiliations:** 1 Department of Molecular Medicine, University of Pavia, Via Carlo Forlanini 16, 27100 Pavia, Italy; 2 Unit of Anatomic Pathology, IRCCS San Matteo Hospital Foundation, Pavia, Italy; 3 Institute of Radiology, IRCCS San Matteo Hospital Foundation, Pavia, Italy; 4 IRCCS Ospedale Policlinico San Martino, Genoa, Italy; 5 Pathology Unit, Department of Surgical Sciences and Integrated Diagnostics (DISC), University of Genoa, Genoa, Italy; 6 Deparment of Internal Medicine and Medical Therapy Department, University of Pavia, Pavia, Italy; 7 Medical Oncology Unit, IRCCS San Matteo Hospital Foundation, Pavia, Italy; 8 Division of General Surgery 1, Fondazione IRCCS Policlinico San Matteo, Pavia, Italy; 9 Department of Clinical, Surgical, Diagnostic and Pediatric Sciences, University of Pavia, Pavia, Italy; 10 Division of General Surgery 2, Fondazione IRCCS Policlinico San Matteo, Pavia, Italy; 11 First Department of Internal Medicine, IRCCS San Matteo Hospital Foundation, Pavia, Italy

**Keywords:** Adenomyomatous hyperplasia, Dysplasia, ERBB2, Intracholecystic papillary neoplasm, HER2 amplification, Gallbladder

## Abstract

Adenomyoma/adenomyomatosis (AM) of the gallbladder is generally considered an incidental and innocuous finding; however, neoplastic lesions, including intracholecystic neoplasms (ICNs), flat-type dysplasia, and carcinomas, may arise within AM. AM-associated ICNs, composed of mural cystically dilated glands containing florid papillary proliferations lined by mucinous and/or overtly dysplastic epithelium, are very rare and poorly characterized. This study aimed at investigating the clinico-radiologic, phenotypic/immunophenotypic, and molecular features of a mono-institutional case series of four AM-ICNs (0.2% of cholecystectomies). Immunohistochemistry for CDX2, MUC2, MUC5AC, MUC6, MUC1, HER2, ß-catenin, and p53, as well as next-generation sequencing of 110 tumor-related genes (AmoyDx® Comprehensive Panel), were performed. Our study confirms the AM-ICN-associated clinico-demographic characteristics previously described, including the relatively low frequency of associated invasive carcinoma (one case, 25%), although high-grade dysplasia (HGD) was observed in three out of four cases. In two cases, imaging findings suspicious for neoplasm were seen. Segmental-type AM was seen in two cases. Predominantly cell phenotype was gastric foveolar in two AM-ICNs and pancreatobiliary in the other two cases (both with HGD), while the immunophenotype was hybrid/mixed in all cases. No case had nuclear ß-catenin expression nor Wnt pathway or *KRAS* gene alterations. One case showed both *HER2* point mutation and *HER2* amplification, while the AM-ICN associated with an invasive adenocarcinoma harbored *TP53* mutation and p53 overexpression. In conclusion, our findings suggest the separation of AM-ICNs from other gallbladder dysplastic lesions.

## Introduction

Adenomyoma (AM), also known as adenomyomatous nodule or adenomyomatosis, is a relatively frequent gallbladder lesion, as it has been found in about 9% of totally submitted and examined cholecystectomies; however, some small AMs may be undiagnosed due to the lack of recognition during gross examination of the surgical specimen [1]. AM is characterized by a mural collection of cystically dilated glands forming a small solitary mass or a band of trabeculated thickening of the gallbladder wall with sieve-like configuration [1, 2]. According to the extent of gallbladder involvement, this lesion may be subdivided into three forms: (i) the localized form, which is the most common and most often found in the fundic region; (ii) the segmental form; and (iii) the diffuse form, which is very rare (about 1% of all AM cases) [1, 2]. Although in the literature AM has also been referred to as “adenomyomatous hyperplasia”, recent findings indicate that AM is most likely a malformative developmental lesion, rather than a growth of hyperplastic or neoplastic nature [1].

Although it is generally considered an incidental and innocuous finding, AM itself can develop dysplastic transformation and might be a source of more invasive gallbladder carcinomas, more so than currently thought [3, 4]. Nevertheless, the frequency of neoplastic transformation of AMs is still controversial, with some studies claiming a greater risk for the segmental form [4, 5]. In a recent investigation, neoplastic changes have been found in almost 10% of AMs (9.9%, 28 out of 183 AMs) [1], indicating that AMs should be grossly searched for and carefully examined histologically in all surgically resected gallbladders.

In addition to flat-type high-grade dysplasia/carcinoma in situ (HGD/CIS) and invasive carcinomas (found in 2.5% and 1.8% of AMs, respectively, by Dursun et al. [1]), AMs may also form papillary tumoral intraepithelial neoplasia or intracholecystic neoplasms (ICNs) (by definition exclusively confined to the AM with preservation of the surface mucosa), which has been only recently well characterized and named “adenomyomatous nodule-associated mural ICNs (AM-ICN)” [6, 7]. The prevalence of AM-ICNs has been estimated to be 0.1% of cholecystectomies. AM-ICNs represent a distinct entity among gallbladder neoplasms, showing the following similarities with pancreatic branch-duct type intraductal papillary mucinous neoplasms (IPMNs): (i) median patient age at diagnosis is over 65 years, (ii) the lack of female predominance typical of many gallbladder diseases, (iii) the frequent gastric-type differentiation, and (iv) the relatively low rate (about 15%) of associated invasive carcinoma [6]. Importantly, AM-ICNs do not show the field-effect/field-defect phenomenon with widespread dysplastic changes of the biliary tract typically observed in ICNs of the gallbladder surface, with different follow-up implications. However, molecular profiles and radiologic features of AM-ICNs are currently unknown.

The present study is aimed at characterizing the clinico-pathologic, radiologic, and molecular features of a mono-institutional case series of AM-ICNs.

## Materials and methods

### Case selection

We searched for AM-ICNs among cholecystectomy specimens from the in-house pathology files at the Departments of Pathology of IRCCS San Matteo Hospital (Pavia, Italy) over a period of 5 years (from August 2019 to August 2024), using the following keywords: [“dysplasia” or “intracholecystic neoplasia”] and [“adenomyoma” or “adenomyomatous hyperplasia”]. Hematoxylin and eosin (H&E) slides of selected cases were reviewed by two pathologists with special interest in biliary pathology (AV and GDA). As proposed by Rowan et al. [6], AM-ICN was histologically defined as follows: a gallbladder AM composed of mural cystically dilated glands containing variable amounts of florid papillary proliferations lined by mucinous and/or overtly dysplastic epithelium. One case has already been included in a previous study [8], but with no in depth molecular analysis and with limited immunohistochemical profiling, while none of the other tumors has been previously reported.

### Collection of clinical information and imaging assessment

The clinical and demographic data of AM-ICN patients were collected using medical charts and included the following: patient age at cholecystectomy, sex, clinical presentation before cholecystectomy, and follow-up data. Gallbladder imaging features on ultrasound (US), computed tomography (CT), and/or magnetic resonance imaging (MRI) were recorded and CT and MRI scans, when available, were also reviewed by an expert abdominal radiologist (AG). Presence of cholelithiasis and AM type, site, and size were assessed, combining both imaging and gross appearance. Specifically, AM type was classified as localized, annular, segmental, or diffuse, as previously reported [9].

### Histopathologic analysis

Dysplasia was graded according to the two-tiered grading system used for biliary intraepithelial neoplasia (i.e., low-grade or high-grade), while predominant cell lineage of AM-ICNs was classified based on morphology as gastric-type, intestinal-type, or pancreatobiliary (PB)-type, as previously reported [6]. If an invasive carcinoma associated with the AM-ICN was detected, its histologic type, size, and stage were evaluated. The gallbladder surface mucosa was also assessed for the presence of metaplastic and/or dysplastic alterations.

### Immunohistochemistry and HER2/ERBB2 fluorescence in situ hybridization

Formalin-fixed paraffin-embedded (FFPE) tissue blocks were retrieved for all AM-ICN cases and immunohistochemical analyses were performed on 3-μm sections cut from the most representative FFPE blocks using a fully automated system (BenchMark Ultra; Ventana Medical Systems, Tucson, AZ, USA) and the following antibodies: MUC1 [clone E29, Roche, prediluted], MUC5AC [clone MRQ-19, Cell Marque, prediluted], MUC6 [clone MRQ-20, Cell Marque, prediluted], MUC2 [clone MRQ-18, Cell Marque, prediluted], and CDX2 [clone EPR2764Y, Cell Marque, prediluted]. These cell lineage markers were regarded as positive when they were expressed in > 5% of neoplastic cells [10]. In addition, immunohistochemistry (IHC) for HER2 [clone 4B5, Roche, prediluted], β-catenin [clone 14, Cell Marque, prediluted], and p53 [clone DO-7, Roche, prediluted] was performed. HER2 IHC was scored according to gastric cancer criteria [11, 12] and nuclear expression of β-catenin in > 5% of neoplastic cells was considered abnormal, while expression of p53 was scored as wild-type (only a few positive cells, with variable intensity) or aberrant (including the complete absence of staining in neoplastic cell nuclei with appropriately staining internal control, and the overexpression pattern, i.e., ≥ 60% of neoplastic cell nuclei strongly positive) [13].

Fluorescence in situ hybridization (FISH) for *HER2/ERBB2* gene amplification was performed in all AM-ICNs and a case was considered *HER2*-amplified when the *HER2*/CEP17 ratio was ≥ 2 [11, 12].

### Next-generation sequencing analysis

We analyzed genetic alterations in 110 tumor-related genes by means of a commercial solution (AmoyDx® Comprehensive Panel, Amoy Diagnostics, Singapore), a next-generation sequencing (NGS)-based assay intended for the qualitative detection of single nucleotide variants (SNVs), insertions and deletions (InDels), gene fusions, copy number variations (CNVs), and microsatellite instability (MSI) status.

The AM-ICN-specific areas were marked on the H&E slides for nucleic acid extraction. Automated extraction and purification of genomic DNA and RNA from FFPE tissue samples was performed, scraping with a clean razor blade specifically selected areas from 4 slides (thickness 8 µm) (Maxwell® CSC DNA FFPE Kit and Maxwell® CSC RNA FFPE Kit, Promega Corporation, USA). Sequencing strategy was performed using the targeted multiplexed capture-based approach starting from genomic DNA; the resulting libraries were sequenced on Illumina platform (NextSeq1000/2000, Illumina, Inc., USA).

Primary analysis of sequencing data was performed by using AmoyDx® NGS data analysis system (ANDAS, Amoy Diagnostics, Singapore), while secondary and tertiary data analysis was performed using a homemade multi-step classification algorithm. Briefly, variants with a variant allele frequency (VAF) lower than 0.03 and/or variants with a coverage < 300 × were filtered out. Single nucleotide polymorphisms (SNP), silent variants, and variants functionally annotated as neutral or likely neutral were then also excluded, based on the information retrieved from public databases [dbSNP (https://www.ncbi.nlm.nih.gov/snp/), gnomAD (https://gnomad.broadinstitute.org/), VarSome (https://varsome.com/), OncoKB (https://www.oncokb.org/), ClinVar (https://www.ncbi.nlm.nih.gov/clinvar/)]. The remaining variants were considered possible somatic mutations and their pathogenic value was evaluated in order to differentiate known and putative pathogenic mutations from variants of unclear significance (VUS).

All variants (missense, in-frame insertions/deletions, frameshift, nonsense, and splice site) were considered pathogenic/possibly pathogenic if they were previously reported in the publicly accessible repository (COSMIC, https://cancer.sanger.ac.uk/cosmic) at least in more than two biliary tract neoplasms. Truncating mutations (nonsense, frameshift, and splice site) were considered possibly pathogenic. Missense variants/in-frame insertions/deletions not fulfilling previous criteria, functionally annotated as oncogenic or possibly oncogenic, were considered pathogenic (OncoKB, ClinVar). The remaining nonsynonymous variants were individually assessed based on the available data from COSMIC and their predicted level of evidence according to AMP guidelines by using automatic VarSome somatic variant classifier (VarSome Premium). Only gene fusions and CNVs functionally annotated as oncogenic or possibly oncogenic were considered pathogenic (OncoKB).

## Results

### Clinical and imaging features

Among 2048 cholecystectomies, five selected cases were reviewed. One was excluded because it did not fulfill the diagnostic criteria of AM-ICN lacking florid papillary architecture and was classified as a flat-type HGD/CIS arising in an AM. Four cases of AM-ICNs were finally identified, accounting for 0.2% of cholecystectomies.

The main clinico-pathologic features of AM-ICNs are summarized in Table 1. Median patient age at diagnosis was 70.5 years (ranging from 49 to 72 years) and the male-to-female ratio was 1:1. Three patients (cases #1, #2 and #4) were asymptomatic, while one (case #3) had upper right quadrant abdominal pain*.*

**Table 1:** Clinico-pathologic, imaging, and molecular features of the four cases of adenomyoma/adenomyomatosis-associated intracholecystic neoplasm

Case number	Age (years)	Sex	Symptoms	Main radiologic findings	AM (type; site,; size)	Grade of dysplasia	Phenotype of dysplasia (predominant pattern; secondary pattern, if present)	MUC1 IHC results	MUC5AC IHC results	MUC6 IHC results	MUC2 IHC results	CDX2 IHC results	HER2 IHC score	HER2 amplification (FISH analysis)	p53 IHC aberrant pattern	NGS (pathogenic molecular alterations; MSI status)	Associated invasive carcinoma	Status at follow-up (months after diagnosis)
Case #1	72	M	None	CEUS: pathological enhancement in the arterial phase of the fundus walls; cholelithiasis CECT: hourglass gallbladder with likely segmental AM	Segmental; distal corpus and fundus; 6 cm	LGD	Gastric	+ (80%)	+ (70%)	+ (80%)	-	+ (10%)	2 +	No	No	No alterations; MSS (neoplastic cell %: 20)	No	Alive without recurrence (19)
Case #2	72	M	None	CECT: AM with enhanced solid intracystic components MRI: intracystic T2-hypointensity; MR-DWI restriction	Localized; fundus; 1.5 cm	HGD	PB	+ (100%)	-	-	-	+ (25%)	3 +	Yes	No	*ERBB2*(NM_004448.3): c.2305G > T p.(D769Y), VAF 64.58% *ERBB2* CNV (gain), copy number 7.07; MSS (neoplastic cell %: 70)	No	Dead of other cause (20)
Case #3	49	F	Right upper quadrant abdominal pain	US: cholelithiasis; possible AM	Segmental; distal corpus and fundus; 6 cm	HGD	PB; intestinal	+ (80%)	+ (10%)	+ (40%)	-	+ (70%)	1 +	No	Yes (overexpression)	*TP53*(NM_000546.5): c.736A > G p.(M246V), VAF 11.20%; MSS (neoplastic cell %: 20)	Yes (pT2aN0)	Alive without recurrence (6)
Case #4	69	F	None	US: cholelithiasis; possible AM	Diffuse; entire gallbladder 8 cm	LGD (predominant) HGD (focal)	LGD: gastric; intestinal HGD: intestinal	LGD: + (60%) HGD: -	LGD: + (60%) HGD: -	LGD: + (60%) HGD: -	LGD: - HGD: -	LGD: + (40%) HGD: + (90%)	0	No	No	NA	No	Alive without recurrence (17)

Legend: *AM* adenomyoma/adenomyomatosis, *CECT* contrast-enhanced computed tomography, *CEUS* contrast-enhanced ultrasound, *CNV* copy number variation, *F* female, *FISH* fluorescence in situ hybridization, *HGD* high-grade dysplasia, *IHC* immunohistochemical, *LGD* low-grade dysplasia, *M* male, *MRI* magnetic resonance imaging, *MR-DWI* magnetic resonance diffusion-weighted imaging, *MSI* microsatellite instability, *MSS* microsatellite stable status, *NA* not available, *NGS* next-generation sequencing, *PB* pancreato-biliary, *US* ultrasound, *VAF* variant allele frequency

Imaging review was possible for only two cases (case #1 and #2), because the other two patients (cases #3 and #4) had only trans-abdominal US examinations, revealing cholelithiasis and thickened gallbladder walls with intramural anechoic cystic spaces, indicative of AM; in the latter cases, unfortunately, no US photo was taken.

**Fig. 1:**
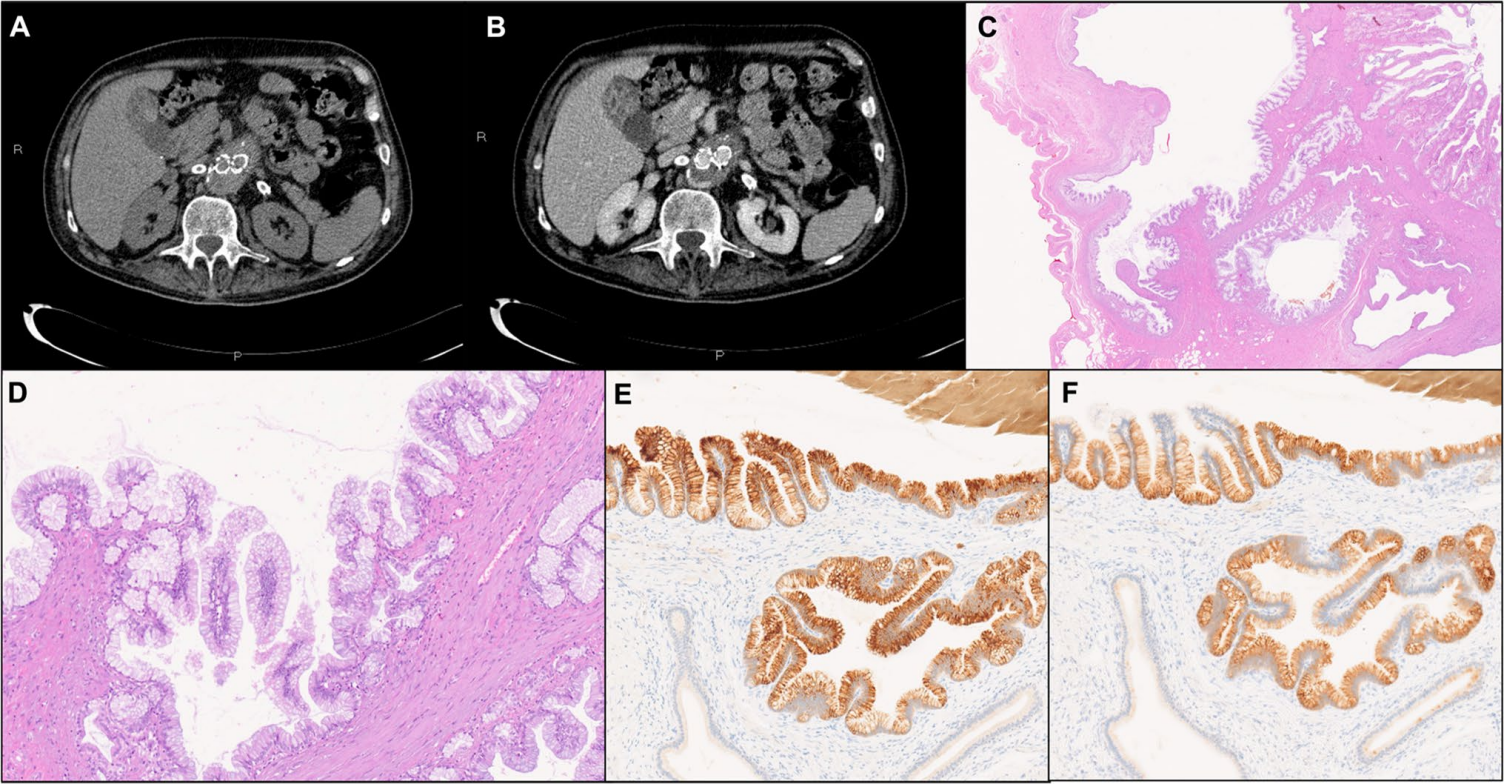
AM-ICN: case #1. **A**, **B** The CT scan shows a dysmorphic gallbladder with markedly thickened corpus and fundus walls containing intramural cystic spaces and a hyperdense component (**A**), without obvious enhancement after contrast medium (**B**). These imaging findings are compatible with a segmental-type AM. **C** Low-power histologic view of the lesion showing the overall architecture of an AM, with cystic structures containing variable amounts of papillary proliferations. Note the normal surface mucosa on the right and the serosa on the left. **D**–**F** At higher magnification, the papillary structures are lined by gastric-type columnar mucinous epithelium with mild atypia (**D**). The mucinous cells are immunoreactive for MUC5AC (**E**) and MUC6 (**F**). **C**, **D** hematoxylin and eosin; **E** MUC5AC immunostaining; **F** MUC6 immunostaining

In case #1, an initial trans-abdominal US showed cholelithiasis and markedly thickened gallbladder wall, followed by contrast-enhanced US (CEUS), that revealed pathological contrast enhancement in the arterial phase of the fundus walls which appeared thickened and irregular; in the portal and late phase, wash-out of the walls was observed. These CEUS findings were considered “suspicions of malignancy” and therefore, the patient also underwent contrast-enhanced CT, which featured a dysmorphic, “hourglass” gallbladder; the gallbladder corpus and fundus walls were markedly thickened and contained large intramural cystic spaces and a hyperdense component, without obvious enhancement, while the gallbladder neck was normal (Fig. 1).

**Fig. 2:**
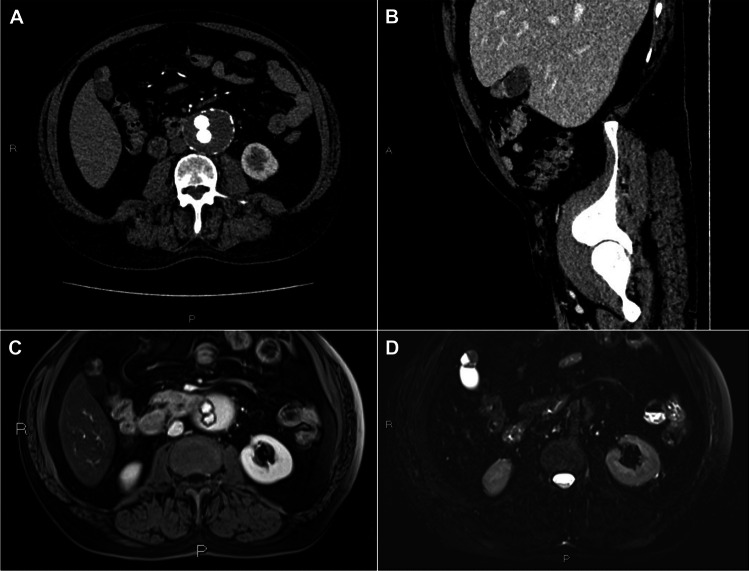
AM-ICN: case #2 imaging features. A CT scan shows arterial enhancement of solid components within mural cystic spaces of the gallbladder fundus (axial plane, **A**), without washout in the venous phase (sagittal plane, **B**), confirmed in the portal phase of the MRI (**C**). In the axial fat-suppressed T2-weighted image, the solid tissue is markedly hypo-intense compared to the adjacent fluid component (**D**). These imaging findings are suspicious for malignancy

Case #2, previously described radiologically by Sanvito et al. [8], had a contrast-enhanced CT, performed after positioning a vascular prosthesis, showing mural cystic spaces of the gallbladder fundus within an unenhanced wall (the so-called “rosary sign”) consistent with AM, as well as an enhancing solid tissue component within some of such cysts (Fig. 2). Therefore, the patient underwent an abdominal MRI which confirmed the presence of intracystic solid areas, which were hypointense on the T2-weighted sequence and spontaneously hyperintense on the T1-weighted sequence, with early contrast enhancement in the arterial phase and no evidence of wash-out in portal- or late-phases. Moreover, a high MR-diffusion-weighted imaging (MR-DWI) signal was documented, indicative of restriction of water molecule movements, which corresponded to hypointensity on the apparent diffusion coefficient (ADC) map.

AM was classified as localized-type in case #2, segmental-type in 2 cases (case #1 and #3), and as diffuse-type in the remaining case (case #4). All patients but one (case #2) showed cholelithiasis.

At follow-up, three patients were alive without evidence of disease recurrence, including the patient (cases #3) who underwent completion cholecystectomy with regional lymphadenectomy without residual neoplasia, whereas patient corresponding to case #2 died of other causes (idiopathic myelofibrosis).

### Histopathologic and immunohistochemical findings

**Fig. 3:**
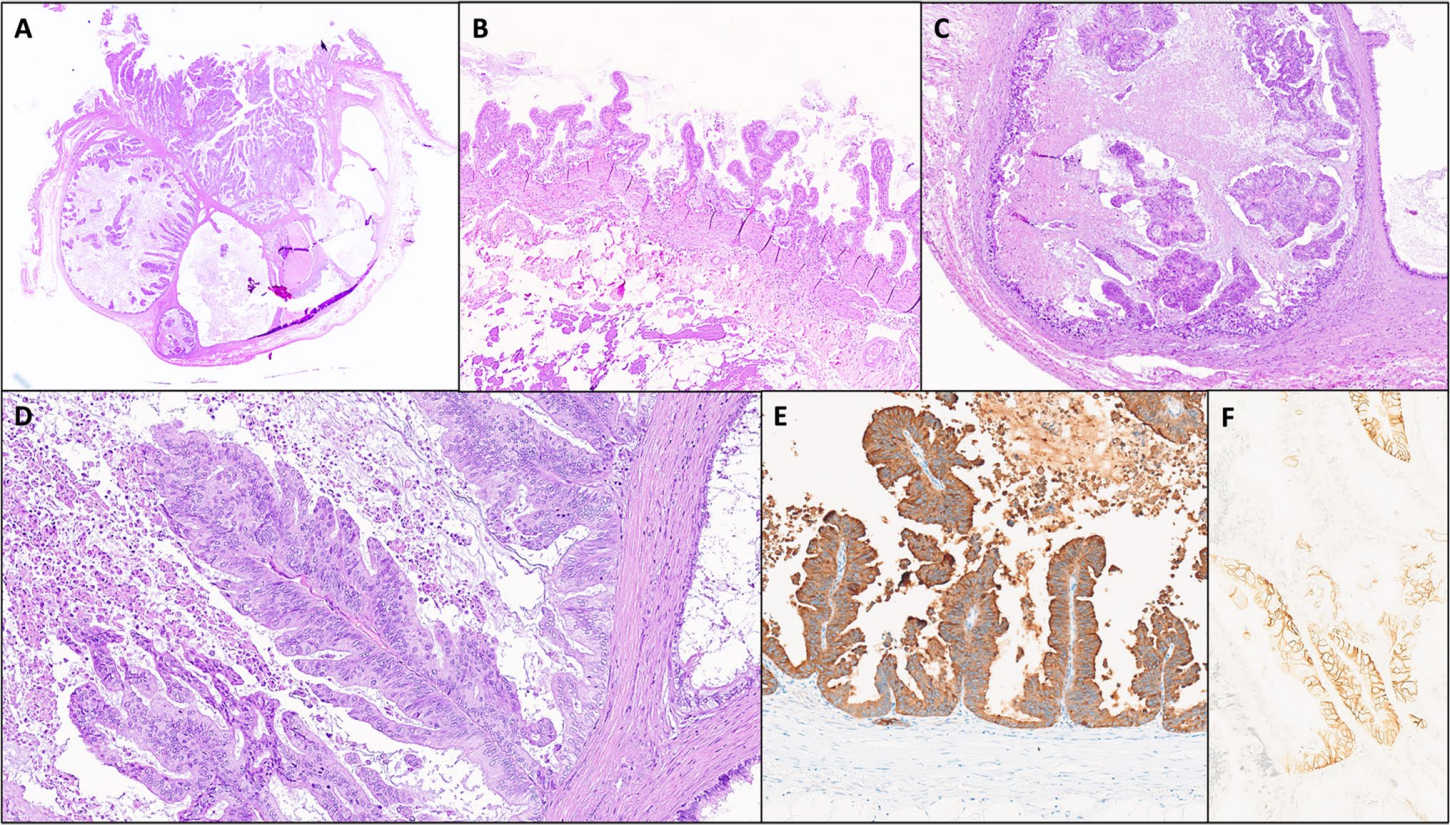
AM-ICN: case #2 histologic features. **A** Low-power histologic view of case #2, showing a localized-type fundus AM, with intramural cysts containing dysplastic papillary proliferations partially extending to the overlying mucosa. **B** The gallbladder mucosa on the edges of the lesion is completely normal. **C** The intracystic papillary growth is florid, with detached papillae within the cystic lumen. **D** At higher magnification, the papillary structures are lined by predominantly cuboidal, biliary-type epithelium, showing eosinophilic cytoplasm and loss of nuclear polarity consistent with high-grade dysplastic features. **E** The dysplastic cells show diffuse expression of MUC1. **F** Strong membranous (complete, lateral, or basolateral) expression of HER2 in > 10% of neoplastic cells (score 3 +) was seen. **A**–**D** hematoxylin and eosin; **E** MUC1 immunostaining; **F** HER2 immunostaining

**Fig. 4:**
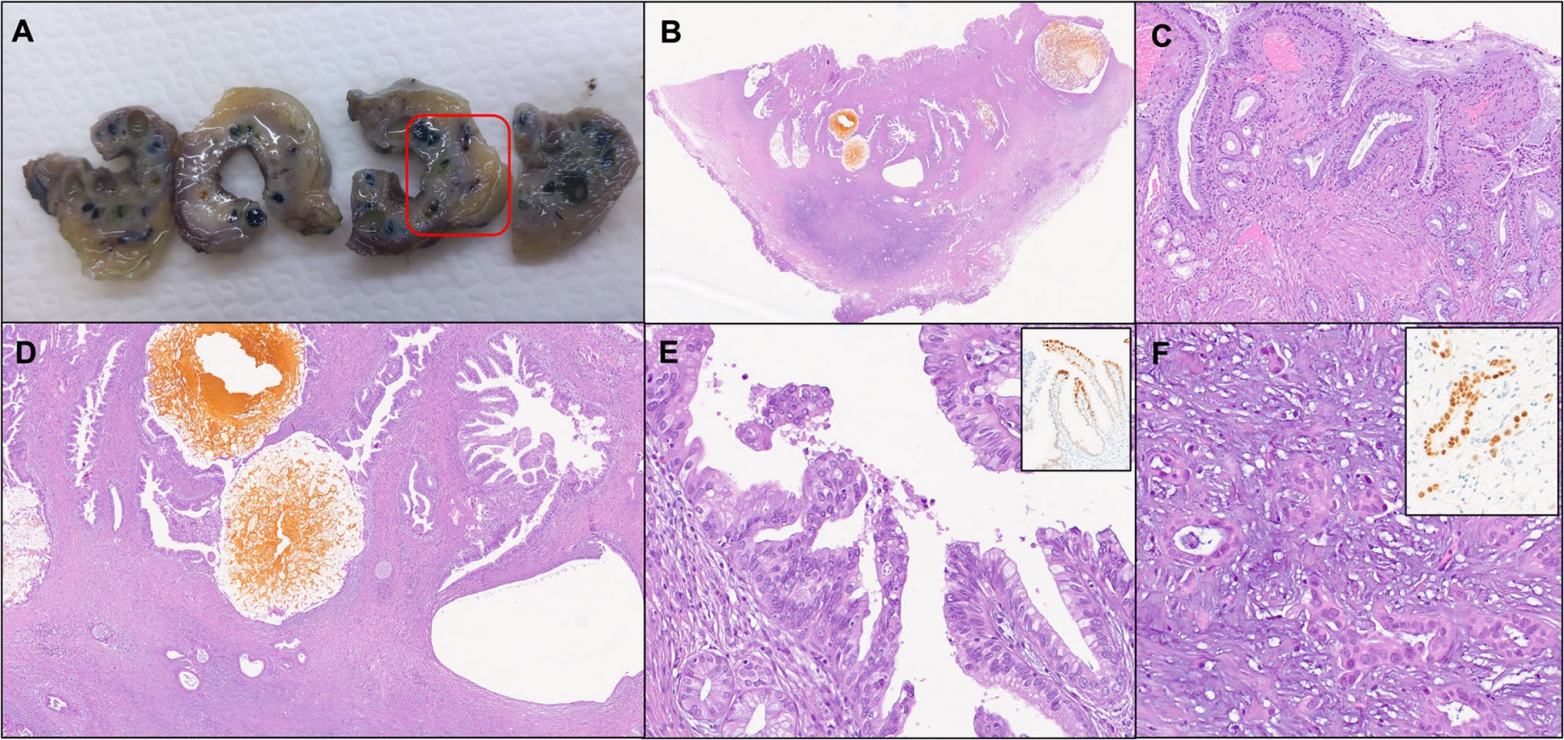
AM-ICN: case #3 macroscopic and microscopic findings. **A** Gross appearance of gallbladder corpus and fundus, showing trabeculated thickening of the walls with a sieve-like configuration, due to the presence of intramural cystic spaces. The red box encircles the area which microscopically corresponds to an invasive adenocarcinoma. **B** The low-power histologic view of the gallbladder wall section within the rex box with a three-layer appearance. **C** The inner layer is represented by the non-dysplastic surface mucosa, with signs of chronic injury, such as pseudopyloric metaplasia. **D** The intermediate and outer layers are composed of AM with cystic structures containing florid papillary growths and of invasive adenocarcinoma in subserosal tissues, respectively. **E** At higher magnification, the intracystic papillary structures are lined by a predominantly biliary-type epithelium with high-grade dysplasia (on the left), with minor foci of intestinal-type differentiation (on the right), as confirmed by CDX2 expression by epithelial cells (inset of **E**). **F** The biliary-type invasive adenocarcinoma is associated with marked desmoplastic reaction and tumor cells show p53 overexpression (inset of **F**). **B, C, D, E, F**: hematoxylin and eosin; inset of **E**: CDX2 immunostaining; inset of **F**: p53 immunostaining

Dysplasia was graded as high-grade (HGD) in three cases (Figs. 3 and 4) and as low-grade (LGD) in one case (case #1, Fig. 1); however, in one case (case #4), both LGD and HGD foci were observed.

The cell lineage was predominantly PB in two cases (cases #2 and #3) and gastric-type in the remaining two cases (cases #1 and #4). Specifically, in case #4, LGD showed gastric foveolar-type predominant differentiation (with minor intestinal-type foci) while the HGD component was of intestinal-type. Some intestinal-type foci were also observed in case #3. The IHC co-expression of intestinal (CDX2) and gastro-PB (MUC1, MUC5AC, and/or MUC6) markers was seen in all AM-ICN cases, albeit with variable proportions, indicating their hybrid immunophenotypes (Table 1).

In case #3, a focus of invasive biliary-type adenocarcinoma (size: 12 mm, stage pT2a) associated with the AM-ICN was identified (Fig. 4), showing the same mixed immunoprofile of the corresponding AM-ICN (co-expression of MUC1, CDX2, and MUC6).

HER2 IHC staining showed score 3 + in one case (case #2) (Fig. 3F), which harbored *HER2/ERBB2* amplification by FISH (*HER2* signals in clusters), while the other cases were *HER2* FISH negative, although one case was scored 2 + (case #1) and another 1 + (case #3) by HER2 IHC (Table 1). In case #3, the associated invasive adenocarcinoma was scored 2 + by HER2 IHC; however, no *HER2* amplification was found by FISH (*HER2*/CEP17 ratio was 1.84).

No β-catenin nuclear staining was seen in any case, while p53 IHC was aberrant in only one case (case #3), which showed p53 overexpression in both the HGD and the invasive adenocarcinoma components.

The surface gallbladder mucosa was unremarkable in two cases (cases #1 and #4). In case #2, the surface mucosa on the edges of and away from the lesion was normal (Fig. 3); however, a focal protrusion of HGD from the AM onto the overlying mucosa was seen. In case #3, gallbladder background mucosa showed some signs of chronic injury (pseudopyloric and intestinal metaplasia) without dysplasia (Fig. 4).

### NGS sequencing results

NGS sequencing results are summarized in Table 1. Genomic DNA and RNA of case #4 were too degraded for NGS sequencing (probably due to hyperfixation), so no molecular analysis was carried out. No gene fusions or MSI were detected. No alterations in *CTNNB1*, *APC*,* STK11*,* KRAS*, or *GNAS* were observed.

Case #1 showed no genomic alterations, while in case #2, two *HER2/ERBB2* alterations with clinical significance were identified: (i) a SNV that occurs in the exon 19 of the gene and in the kinase functional domain of the protein [ERBB2(NM_004448.3):c.2305G > T p.(D769Y)] and (ii) a CNV, gain-type.

Finally, a pathogenic SNV in *TP53* occurring in exon 7 of the gene and in the functional DNA-binding domain (DBD) of the protein was found in case #3 [TP53(NM_000546.5):c.736A > G p.(M246V)].

## Discussion

In the present study, we have investigated from a clinical-pathological and molecular point of view four cases of AM-ICN, a rare intraepithelial neoplastic lesion of the gallbladder. This is the first work that has molecularly characterized AM-ICNs.

In our case series, we have confirmed the previously described AM-ICN-associated clinico-demographic characteristics [6, 14], including older age at diagnosis, lack of female prevalence, and relatively low frequency of associated invasive carcinoma (1 out of 4 cases, 25% in our series). No recurrence or dysplastic/neoplastic lesions in the remainder of the bile duct system were seen in any case; nevertheless, the median follow-up time was short (median: 18 months).

Our study confirms that the clinical and radiological diagnosis of AM-ICNs is challenging. Worth noting is that three (75%) of our AM-ICN patients were asymptomatic and performed radiologic evaluation for other reasons*.* Although AM-ICN radiologic features remain poorly known, our findings highlight the potential role of CEUS, contrast-enhanced CT, and MR in the detection of neoplastic changes arising within AMs, as previously suggested [8, 15]. Indeed, a neoplastic lesion was radiologically suspected in two out of four cases by CEUS, CT, and/or RM. Importantly, even though further confirmatory studies are required, MR-DWI restriction, coupled with the ADC map evaluation, may prove helpful to detect or rule out the presence of carcinomatous transformation within an AM, in keeping with previous studies indicating that the use of ADC in DWI can improve diagnostic performance in the distinction between malignant and benign gallbladder diseases [16, 17].

Histologically, HGD was identified in most cases (3 out of 4 AM-ICNs, 75%), while Rowan et al. identified HGD in a lower fraction of cases (7 out 19 cases, 37%) [6]. This discrepancy may be possibly due to the limited number of cases in our study and/or to our inclusion of all types of AMs, including segmental-type, which has been associated with a higher risk of gallbladder cancer development in the literature [1, 5, 18]. Indeed, two (50%) AM-ICNs here examined arose in segmental-type AMs and one in diffuse-type AMs, both of which are much rarer than localized-type AMs [1]. Due to the radiologic difficulties in detecting carcinomatous transformation of AMs, prophylactic cholecystectomy or close surveillance imaging could be considered for asymptomatic segmental-type and diffuse-type AMs, as previously suggested [4, 19], as well as for AMs with atypical imaging features.

Interestingly, all AM-ICNs showed a hybrid or complex immunophenotype in our study, similar to most intracholecystic papillary-tubular neoplasms of the surface mucosa (Sur-ICPNs) [9]; in addition, MUC1 and CDX2 were expressed in all cases, albeit in variable proportions. In one case, where both LGD and HGD components were recognizable, the LGD areas were of gastric-type whereas the HGD was of intestinal-type. These findings suggest that AM-ICN cell lineage transitions may occur during neoplastic progression.

Although the small number of cases analyzed may not be representative of the molecular features of AM-ICNs as a whole, and larger multicenter studies are needed to draw definitive conclusions and make comparisons with other ICNs, there are some interesting insights worth noting to stimulate future research. First, no nuclear aberrant expression of β-catenin, a marker of canonical Wnt pathway activation, and no *CTNNB1*, *APC*, *or STK11* mutation were found in the three AM-ICNs investigated in this study, suggesting a limited role of the Wnt pathway in AM-ICN development. Second, one AM-ICN of our study harbored both *HER2* amplification (with HER2 IHC score 3 +) and a *HER2* point mutation. In addition, two other cases showed HER2 IHC score 2 + or 1 + , without gene amplification. Our findings may suggest a possible pathogenetic role of *HER2* molecular changes in the development of an AM-ICN subset. *HER2* is considered a driver gene in gallbladder carcinogenesis [20]. Activated *ERBB2/ERBB3* mutations have been reported to promote tumor cell proliferation and migration, as well as increase PD-L1 expression to induce immune evasion, and are associated with worse patient survival [21]. Moreover*, HER2* is an actionable target in multiple cancers, including gallbladder cancer, with a variety of anti-HER2 therapies [21–23]. *HER2* mutation has been observed in only 3–6% of gallbladder carcinomas, while *HER2* amplification has been reported in 5–10% of gallbladder carcinomas and was also described in 4% of HGD of the gallbladder with associated invasive carcinoma [11, 20–22]. Whether and how often the different subtypes of ICNs harbor *HER2* gene alterations remain to be investigated further. Similarly, whether gallbladder carcinomas associated with AMs (with or without AM-ICNs) are associated with a greater frequency of *HER2* alterations compared to the remaining gallbladder cancers is also as yet unknown.

This study has several limitations including the small number of cases and the relatively few genes investigated. In one case, no mutation in examined genes was found, while in another case, molecular analysis was unsuccessful; whole exome sequencing studies on larger series should be able to identify other potential driver mutations involved in AM-ICN development.

To conclude, AM-ICNs are rare dysplastic lesions of the gallbladder, with peculiar clinico-pathologic features, including the lack of female prevalence, the minimal or no involvement of the surface mucosa coupled with the lack of dysplasia in the remainder of the gallbladder (no field-effect phenomenon), and the relatively low frequency of associated invasive carcinoma (though HGD is not uncommon), suggesting their separation from other gallbladder dysplastic lesions. AM-ICNs have often hybrid/mixed phenotypes and immunophenotypes, and a few may harbor *HER2* alterations.

## Data Availability

The datasets generated during and/or analyzed during the current study are available from the corresponding author on reasonable request.

## References

[1] Dursun N, Memis B, Pehlivanoglu B, Taskin OC, Okcu O, Akkas G, Bagci P, Balci S, Saka B, Araya JC, Bellolio E, Roa JC, Jang KT, Losada H, Maithel SK, Sarmiento J, Reid MD, Jang JY, Cheng JD, Basturk O, Koshiol J, Adsay NV (2024) Adenomyomas of the gallbladder: an analysis of frequency, clinicopathologic associations, and relationship to carcinoma of a malformative lesion. Arch Pathol Lab Med 148:206–214. 10.5858/arpa.2022-0379-OA 37134225 10.5858/arpa.2022-0379-OA PMC12301898

[2] Basturk O, Adsay NV (2022) Benign and malignant tumors of the gallbladder and extrahepatic biliary tract. In: Odze RD, Goldblum JR (eds) Surgical Pathology of the GI Tract, Liver, Biliary Tract and Pancreas, IV. Elsevier, Philadelphia, pp 1197–1235

[3] Albores-Saavedra J, Shukla D, Carrick K, Henson DE (2004) In situ and invasive adenocarcinomas of the gallbladder extending into or arising from Rokitansky-Aschoff sinuses: a clinicopathologic study of 49 cases. Am J Surg Pathol 28:621–628. 10.1097/00000478-200405000-00009 15105650 10.1097/00000478-200405000-00009

[4] Kai K, Ide T, Masuda M, Kitahara K, Miyoshi A, Miyazaki K, Noshiro H, Tokunaga O (2011) Clinicopathologic features of advanced gallbladder cancer associated with adenomyomatosis. Virchows Arch 459:573–580. 10.1007/s00428-011-1155-1 22038508 10.1007/s00428-011-1155-1

[5] Ootani T, Shirai Y, Tsukada K, Muto T (1992) Relationship between gallbladder carcinoma and the segmental type of adenomyomatosis of the gallbladder. Cancer 69:2647–2652 1571894 10.1002/1097-0142(19920601)69:11<2647::aid-cncr2820691105>3.0.co;2-0

[6] Rowan DJ, Pehlivanoglu B, Memis B, Bagci P, Erbarut I, Dursun N, Jang KT, Sarmiento J, Mucientes F, Cheng JD, Roa JC, Araya JC, Bellolio E, Losada H, Jang JY, Koshiol J, Reid MD, Basturk O, Adsay V (2020) Mural intracholecystic neoplasms arising in adenomyomatous nodules of the gallbladder: an analysis of 19 examples of a clinicopathologically distinct entity. Am J Surg Pathol 44:1649–1657. 10.1097/PAS.0000000000001603 33060404 10.1097/PAS.0000000000001603 PMC7658044

[7] Roa JC, Basturk O, Adsay V (2021) Dysplasia and carcinoma of the gallbladder: pathological evaluation, sampling, differential diagnosis and clinical implications. Histopathology 79:2–19. 10.1111/his.14360 33629395 10.1111/his.14360

[8] Sanvito F, Gallotti A, Cobianchi L, Vanoli A, Cho NS (2022) Preda L (2022) Magnetic resonance diffusion-weighted imaging for detecting fundal intracholecystic papillary neoplasm inside Rokitansky-Aschoff sinuses: a comparison of two cases and a literature review. Radiation 2:52–61. 10.3390/radiation2010004

[9] Bonatti M, Vezzali N, Lombardo F, Ferro F, Zamboni G, Tauber M, Bonatti G (2017) Gallbladder adenomyomatosis: imaging findings, tricks and pitfalls. Insights Imaging 8:243–253. 10.1007/s13244-017-0544-7 28127678 10.1007/s13244-017-0544-7 PMC5359147

[10] Adsay V, Jang KT, Roa JC, Dursun N, Ohike N, Bagci P, Basturk O, Bandyopadhyay S, Cheng JD, Sarmiento JM, Escalona OT, Goodman M, Kong SY, Terry P (2012) Intracholecystic papillary-tubular neoplasms (ICPN) of the gallbladder (neoplastic polyps, adenomas, and papillary neoplasms that are ≥1.0 cm): clinicopathologic and immunohistochemical analysis of 123 cases. Am J Surg Pathol 36:1279–1301. 10.1097/PAS.0b013e318262787c 22895264 10.1097/PAS.0b013e318262787c

[11] Albrecht T, Rausch M, Roessler S, Geissler V, Albrecht M, Halske C, Seifert C, Renner M, Singer S, Mehrabi A, Vogel MN, Pathil-Warth A, Busch E, Köhler B, Rupp C, Weiss KH, Springfeld C, Röcken C, Schirmacher P, Goeppert B (2020) HER2 gene (ERBB2) amplification is a low-frequency driver with potential predictive value in gallbladder carcinoma. Virchows Arch 476:871–880. 10.1007/s00428-019-02706-6 31838585 10.1007/s00428-019-02706-6

[12] Quaquarini E, Grillo F, Gervaso L, Arpa G, Fazio N, Vanoli A, Parente P (2024) Prognostic and predictive roles of HER2 status in non-breast and non-gastroesophageal carcinomas. Cancers 16:3145. 10.3390/cancers16183145 39335117 10.3390/cancers16183145 PMC11430748

[13] Köbel M, Piskorz AM, Lee S, Lui S, LePage C, Marass F, Rosenfeld N, Mes Masson AM, Brenton JD (2016) Optimized p53 immunohistochemistry is an accurate predictor of TP53 mutation in ovarian carcinoma. J Pathol Clin Res 2:247–258. 10.1002/cjp2.53 27840695 10.1002/cjp2.53 PMC5091634

[14] Adsay NV, Basturk O (2024) Dysplasia and early carcinoma of the gallbladder and bile ducts: terminology, classification, and significance. Gastroenterol Clin North Am 53:85–108. 10.1016/j.gtc.2023.10.001 38280752 10.1016/j.gtc.2023.10.001

[15] Muranushi R, Saito H, Matsumoto A, Kato T, Tanaka N, Nakazato K, Morinaga N, Shitara Y, Ishizaki M, Yoshida T, Aishima S, Shirabe K (2018) A case report of intracholecystic papillary neoplasm of the gallbladder resembling a submucosal tumor. Surg Case Rep 4(1):124. 10.1186/s40792-018-0524-2 30264362 10.1186/s40792-018-0524-2 PMC6160379

[16] Ogawa T, Horaguchi J, Fujita N, Noda Y, Kobayashi G, Ito K, Koshita S, Kanno Y, Masu K, Sugita R (2012) High b-value diffusion-weighted magnetic resonance imaging for gallbladder lesions: differentiation between benignity and malignancy. J Gastroenterol 47:1352–1360. 10.1007/s00535-012-0604-1 22576026 10.1007/s00535-012-0604-1

[17] Kitazume Y, Taura S, Nakaminato S, Noguchi O, Masaki Y, Kasahara I, Kishino M, Tateishi U (2016) Diffusion-weighted magnetic resonance imaging to differentiate malignant from benign gallbladder disorders. Eur J Radiol 85:864–873. 10.1016/j.ejrad.2016.02.003 26971436 10.1016/j.ejrad.2016.02.003

[18] Nabatame N, Shirai Y, Nishimura A, Yokoyama N, Wakai T, Hatakeyama K (2004) High risk of gallbladder carcinoma in elderly patients with segmental adenomyomatosis of the gallbladder. J Exp Clin Cancer Res 23:593–598 15743029

[19] Golse N, Lewin M, Rode A, Sebagh M, Mabrut JY (2017) Gallbladder adenomyomatosis: diagnosis and management. J Visc Surg 154:345–353. 10.1016/j.jviscsurg.2017.06.004 28844704 10.1016/j.jviscsurg.2017.06.004

[20] Giraldo NA, Drill E, Satravada BA, Dika IE, Brannon AR, Dermawan J, Mohanty A, Ozcan K, Chakravarty D, Benayed R, Vakiani E, Abou-Alfa GK, Kundra R, Schultz N, Li BT, Berger MF, Harding JJ, Ladanyi M, O’Reilly EM, Jarnagin W, Vanderbilt C, Basturk O, Arcila ME (2022) Comprehensive molecular characterization of gallbladder carcinoma and potential targets for intervention. Clin Cancer Res 28:5359–5367. 10.1158/1078-0432.CCR-22-1954 36228155 10.1158/1078-0432.CCR-22-1954 PMC9772093

[21] Li M, Liu F, Zhang F, Zhou W, Jiang X, Yang Y, Qu K, Wang Y, Ma Q, Wang T, Bai L, Wang Z, Song X, Zhu Y, Yuan R, Gao Y, Liu Y, Jin Y, Li H, Xiang S, Ye Y, Zhang Y, Jiang L, Hu Y, Hao Y, Lu W, Chen S, Gu J, Zhou J, Gong W, Zhang Y, Wang X, Liu X, Liu C, Liu H, Liu Y, Liu Y (2018) Genomic *ERBB2*/*ERBB3* mutations promote PD-L1-mediated immune escape in gallbladder cancer: a whole-exome sequencing analysis. Gut 68:1024–1033. 10.1136/gutjnl-2018-316039 29954840 10.1136/gutjnl-2018-316039

[22] de Bitter TJJ, de Reuver PR, de Savornin Lohman EAJ, Kroeze LI, Vink-Börger ME, van Vliet S, Simmer F, von Rhein D, Jansen EAM, Verheij J, van Herpen CML, Nagtegaal ID, Ligtenberg MJL, van der Post RS (2022) Comprehensive clinicopathological and genomic profiling of gallbladder cancer reveals actionable targets in half of patients. NPJ Precis Oncol 6:83. 10.1038/s41698-022-00327-y 36335173 10.1038/s41698-022-00327-y PMC9637208

[23] Ten Haaft BH, Pedregal M, Prato J, Klümpen HJ, Moreno V, Lamarca A (2024) Revolutionizing anti-HER2 therapies for extrahepatic cholangiocarcinoma and gallbladder cancer: current advancements and future perspectives. Eur J Cancer 199:113564. 10.1016/j.ejca.2024.113564 38266541 10.1016/j.ejca.2024.113564

